# Mental Health Promotion and Illness Prevention in Vulnerable Populations

**DOI:** 10.3390/healthcare12050554

**Published:** 2024-02-28

**Authors:** Carlos Laranjeira, Ana Querido

**Affiliations:** 1 Centre for Innovative Care and Health Technology (ciTechCare), Polytechnic University of Leiria, Campus 5, Rua das Olhalvas, 2414-016 Leiria, Portugal; ana.querido@ipleiria.pt; 2 School of Health Sciences, Polytechnic University of Leiria, Campus 2—Morro do Lena, Alto do Vieiro—Apart. 4137, 2411-901 Leiria, Portugal; 3 Comprehensive Health Research Centre (CHRC), University of Évora, 7000-801 Évora, Portugal; 4 Center for Health Technology and Services Research (CINTESIS), NursID, University of Porto, 4200-450 Porto, Portugal

Several cases of social and health inequity have occurred in recent centuries. Despite major innovations and significant changes in the general population’s quality of life, the most vulnerable populations continued to survive [1]. Vulnerability is a general concept that, in the context of health, means susceptibility to developing a certain health problem. Thus, vulnerable populations are those at greater risk of developing physical, psychological, or social health problems due to their marginalized socio-cultural status, their limited access to economic resources, or due to individual characteristics such as gender or age [2]. Vulnerability, as a human being’s ontological condition [3,4], can be aggravated by experiencing a transition, exposing the client to potential danger, a problematic or excessively long recovery, or an inadequate or delayed adaptation process [5,6].

Vulnerable groups are those who are socially, psychologically, and/or materially more susceptible to social exclusion due to reasons of health, sexual orientation, religion, culture, ethnicity, gender, physical or mental disability, among others. Thus, they are more likely to develop health problems and needs than the rest of the population. In this vein, vulnerability is also a dynamic and multidimensional process, with interactions between personal characteristics and social and environmental conditions. Many socially explicable risk factors and co-morbidities—such as low socioeconomic status or substandard living conditions—are present in vulnerable groups. Rather than being attributable to personal traits, vulnerability is the product of social processes that may lead to greater exposure to different types of risk, higher sensitivity to negative consequences, and a lower ability to react by coping or adapting [7,8]. Therefore, to tackle health disparities, public health interventions should focus on the underlying social and historical factors that perpetuate poor health in marginalized communities. Frohlich and Potvin [9] offer a broad overview of these interventions, emphasizing that they should be intersectoral and participatory [2,10]. However, further investigation is required to determine the specific requirements for promoting fair outcomes for vulnerable populations.

Ensuring the prevention and promotion of mental health is crucial, particularly to mitigate the increasing prevalence of mental disorders. Although health promotion and disease prevention are well-recognized principles in the field of public health, their effective implementation to promote and prevent mental health is sometimes challenging [11]. Thankfully, there has been significant advancement in recent decades, including substantial study on the subject. Given these advancements, academics, care providers, governments, and policymakers are increasingly focused on using preventative techniques to enhance the availability, accessibility, and effectiveness of these services for the population in a less stigmatizing, culturally congruent, and accessible manner.

This Special Issue covers a diverse array of topics and comprises 23 papers that garnered a total of 29,911 views worldwide before drafting this editorial. The studies encompass different populations, including people with disabilities, women with breast cancer, survivors of humidifier disinfectant damage, patients with hematologic malignancies and candidates for allogeneic hematopoietic stem cell transplantation, individuals with depression, suicidal ideation, and other mental health conditions. Additionally, it includes studies on populations facing psychopathological symptoms, such as general populations, older adults, women facing high-risk pregnancies, postpartum women, prison staff, and other vulnerable people in the community. Moreover, this Special Issue covers carers, including adult children of parents with mental illness, adult-child caregivers, parents, caregivers, and teachers of adolescents in secondary school, caregivers of persons with mental disorders, and health care providers. It also encompasses studies of various student populations, including first-year medical students, college students, and university students.

This Special Issue of papers is a unique contribution to the literature on mental health promotion and illness prevention in vulnerable populations. This broad research topic also educates professionals in the fields of healthcare, education, and social services on the significance and advantages of their activities in enhancing mental health within a population. It encourages them to serve as facilitators, mediators, and advocates for mental health across different sectors. These studies from different cultural backgrounds further illuminate and enhance our understanding of how to assess and recognize populations susceptible to mental and behavioral disorders, and how to foster mental well-being and socio-emotional health across all stages of life (such as children, adolescents, adults, elderly individuals, and other vulnerable groups), providing valuable insights for both scholars and practitioners.
